# Behavioral Interventions for Consumer Food Waste Reduction: A Multi-Level Governance Perspective

**DOI:** 10.3390/foods15162865

**Published:** 2026-08-17

**Authors:** Xia Zhao, Zhenling Zhang

**Affiliations:** Institute of Food and Strategic Reserves, Nanjing University of Finance and Economics, Nanjing 210023, China; 1920250031@stu.nufe.edu.cn

**Keywords:** food waste, consumer behavior, behavioral intervention, choice architecture, multi-level governance, sustainability

## Abstract

Consumer food waste is a major challenge for food system sustainability and a governance problem that cannot be addressed by individual behavioral change alone. Previous reviews have examined the causes of food waste or evaluated specific intervention strategies, but less attention has been paid to how behavioral drivers, intervention strategies, and the roles of governance actors can be examined within an integrated analytical framework. Against this background, this review aims to synthesize the behavioral drivers of consumer food waste, classify the main types of behavioral interventions, and develop a multi-level governance perspective that connects these elements. Using a narrative review approach supported by a structured literature search, the review identifies three broad categories of behavioral drivers, namely cognitive biases, motivational drivers, and environmental and situational influences. It then classifies behavioral interventions into five types, namely information-based interventions, social norm interventions, choice architecture and environmental design interventions, incentive-based interventions, and education and capacity-building interventions. The review indicates that no single intervention type is likely to be effective across all settings. Instead, intervention outcomes are closely linked to the alignment among targeted behavioral drivers, intervention strategies, consumption contexts, and governance support. From a multi-level governance perspective, this review views consumers as the actors engaged in everyday food-related practices, businesses as the designers of the consumption environments and operational routines, and policymakers as the providers of institutional, informational, and economic support. By bringing behavioral drivers, intervention strategies, and governance roles into a shared analytical framework, this review moves beyond fragmented, individual-centered approaches to reducing consumer food waste. The framework suggests that food waste reduction efforts may be more effectively implemented and maintained when consumer practices, business environments, and policy conditions are mutually supportive.

## 1. Introduction

Food waste is considered one of the most challenging sustainability issues of the 21st century, with far-reaching environmental, economic, and social implications. From an environmental perspective, food waste not only leads to the inefficient use of resources such as land, water, and energy, but also exacerbates climate change, biodiversity loss, and ecosystem degradation. According to the 2024 Food Waste Index Report released by the United Nations Environment Programme, global food waste totaled approximately 1.05 billion metric tons in 2022, accounting for almost one-fifth of the total food available to consumers at the retail, food service, and household levels. Greenhouse gas emissions from food waste account for 8% to 10% of global emissions, approximately five times the emissions of the aviation industry. Meanwhile, food waste results in the inefficient utilization of 28% of global agricultural land and 25% of freshwater resources [1]. Economically, food waste causes losses of nearly USD 1 trillion, reduces the efficiency of food systems, and increases resource misallocation and public governance costs [1]. Socially, food waste stands in stark contrast to the fact that more than 700 million people worldwide suffer from hunger [2], further exacerbating the global food crisis and social inequality and weakening the resilience of food systems. Therefore, reducing food waste has become an important issue in global sustainability governance. United Nations Sustainable Development Goal 12.3 explicitly calls for halving per capita global food waste at the retail and consumer levels by 2030 and reducing food losses along production and supply chains, providing an important basis for countries to formulate food waste governance policies and action plans.

Among the various stages of the food supply chain, the consumer stage is particularly important because waste at this stage is both substantial in scale and complex in its causes [3]. Particularly in developed countries, consumer-stage waste accounts for a large proportion of total food waste [4]. In China, the total food loss and waste rate across all stages is approximately 22.7%, amounting to about 460 million tons, with consumption representing one of the most important stages of food loss and waste [5]. In contrast to the production and distribution stages, which are mainly constrained by technology and infrastructure, food waste at the consumer stage arises primarily from individual behavioral decisions made during purchasing, storage, and consumption. These decisions are influenced by a combination of factors, including cognitive biases, motivational drivers, and environmental and situational influences. Therefore, reducing consumer food waste requires not only technical and institutional measures, but also a behavioral understanding of how waste is generated in everyday life.

Traditional policies to reduce food waste have often relied on regulatory constraints and market incentives, but their effectiveness is constrained by implementation conditions. Regulatory measures, such as mandatory donations and anti-food waste laws, may be weakened by ambiguous legal provisions, limited institutional coordination, and insufficient alignment with specific consumption scenarios [6,7,8]. Market incentives such as tax breaks and subsidies can also be constrained by imprecise targeting, imperfect enforcement mechanisms, and uncertain behavioral responses [9,10,11]. These limitations suggest that institutional constraints and market incentives alone are insufficient to address the complexity of consumer behavior. Governance approaches that can be embedded in everyday decision-making environments, operate at relatively low cost, and respond to behavioral mechanisms have therefore become an important direction in food waste governance research.

Behavioral interventions can reshape the decision-making processes that generate consumer food waste. Rather than relying solely on coercive regulation or strong economic constraints, behavioral interventions seek to change behavior by modifying information presentation, social norm cues, choice environments, perceived costs and benefits, and practical conditions for action [12]. Evidence from household, retail, and food service settings suggests that well-designed interventions can curb food waste under appropriate conditions [13,14,15].

Existing reviews have synthesized consumer food waste from several complementary perspectives, including behavioral drivers and household practices [16,17,18], food waste prevention interventions and their effectiveness [19,20], and behavioral or multi-level frameworks that examine how individual, household, social, and broader contextual factors interact to shape food waste outcomes [21,22]. Together, these reviews provide a useful basis for tracing the recent development of the field.

**Figure 1:**
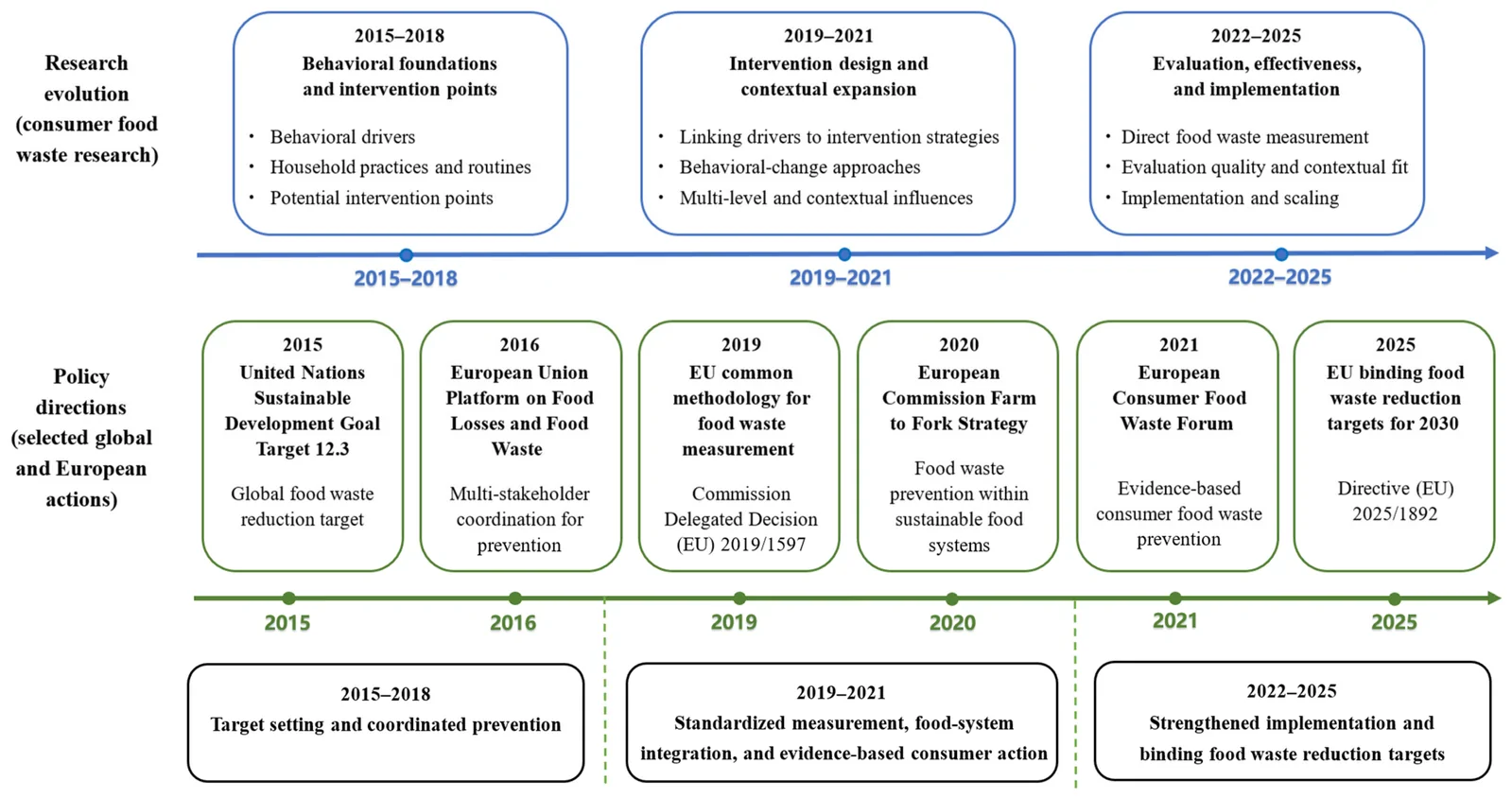
Research and Policy Developments in Consumer Food Waste, 2015–2025.

Viewed over the period from 2015 to 2025, consumer food waste research shows a progressive broadening of emphasis [23]. Earlier research was primarily concerned with understanding why food waste occurs and identifying feasible points for prevention [16,17,18]. Subsequent research increasingly connected behavioral understanding with intervention design [24], while giving greater attention to the wider contexts in which interventions are implemented [21,22]. More recent studies have placed greater emphasis on assessing intervention effectiveness, including through direct measurement of food waste outcomes and greater attention to evaluation quality [19], as well as on the conditions for implementation and scaling [20]. This development broadly parallels global and European food waste governance, which has progressed from target setting and coordinated prevention toward standardized measurement, sustainable food-system integration, evidence-based consumer action, and stronger implementation commitments [25,26,27,28,29,30]. Figure 1 summarizes these parallel developments and selected policy milestones from 2015 to 2025. The comparison indicates broad alignment in evolving priorities rather than a causal relationship between policy developments and changes in research emphasis.

Despite these advances, important gaps remain in how these strands of research are connected. Existing reviews have paid limited attention to integrating behavioral drivers, intervention types, and the conditions under which interventions can be implemented and sustained within a shared analytical framework. Reviews of behavioral drivers often devote less attention to how identified drivers and mechanisms relate to different intervention strategies, while intervention-centered reviews tend to organize evidence by intervention type or effect, with less systematic attention to the behavioral logic underlying these interventions and the conditions that shape their effectiveness. Although behavioral and multi-level perspectives increasingly recognize broader contextual influences, the roles of businesses and policymakers in shaping the conditions under which consumer-level interventions are implemented and sustained are less consistently connected with behavioral drivers and intervention design.

To address this fragmentation, this review develops a multi-level governance perspective on behavioral interventions for consumer food waste reduction. The central argument is that consumer food waste should not be understood solely in terms of individual-level behavioral factors. Rather, food-related practices are shaped by behavioral drivers as well as by business-shaped consumption environments and policy-shaped institutional conditions. Accordingly, behavioral interventions are considered in relation to the behavioral mechanisms they target, the settings in which food-related decisions occur, and the governance conditions that support their implementation.

Guided by this perspective, this review addresses three questions. First, what behavioral drivers contribute to consumer food waste, and through what mechanisms do they operate? Second, what types of behavioral interventions have been used to reduce consumer food waste, and how do their behavioral logics relate to the drivers and mechanisms identified in this review? Third, how can these interventions be supported, implemented, and sustained through the interrelated roles of consumers, businesses, and policymakers? By answering these questions, this review contributes to the literature by connecting behavioral drivers, intervention strategies, and governance roles within an integrated analytical framework for consumer food waste reduction.

To address these questions, this article adopts a narrative review approach to synthesize consumer food waste research through the lens of behavioral interventions. It first organizes the behavioral drivers of consumer food waste into three categories, namely cognitive biases, motivational drivers, and environmental and situational influences. It then classifies behavioral interventions into five types, namely information-based, social norm, choice architecture and environmental design, incentive-based, and education and capacity-building interventions. Building on this synthesis, the article develops a multi-level governance perspective that clarifies how consumers, businesses, and policymakers jointly shape the conditions under which behavioral interventions can be enacted, embedded, and sustained.

## 2. Methodology

This review adopts a narrative review approach supported by a structured literature search. This design is appropriate for integrating dispersed and interdisciplinary evidence, identifying the main behavioral drivers of consumer food waste and the major types of intervention strategies, and developing a multi-level governance perspective, rather than estimating the pooled effects of interventions or conducting an exhaustive systematic review of all relevant studies.

Relevant literature was searched in the Web of Science Core Collection on 30 January 2026. Web of Science Core Collection indexes a large body of peer-reviewed journal literature across food studies, sustainability research, consumer behavior, psychology, economics, sociology, and related fields, making it suitable for identifying core literature on the interdisciplinary topic of consumer food waste. The search covered the Science Citation Index Expanded (SCI-EXPANDED), Social Sciences Citation Index (SSCI), and Emerging Sources Citation Index (ESCI). The Topic field was used to search titles, abstracts, author keywords, and Keywords Plus. Two search blocks were developed and combined using the Boolean operator AND. The first block identified studies concerning food waste at the consumer or household level, while the second covered behavioral drivers and food-waste reduction interventions. The complete search strategy was as follows: TS = ((“food waste” OR “plate waste”) AND (consumer OR household OR customer)) AND TS = ((behavio* OR factor* OR decision OR determinant OR driver OR cognitive OR awareness OR motivation OR convenience OR habit OR routine OR food management) OR (intervention* OR reduc* OR prevent* OR norm* OR information OR nudg* OR incentive OR education)). The search was limited to English-language peer-reviewed journal publications published between January 2010 and January 2026. Conference proceedings, news items, commentaries, letters, and other non-journal publication types were excluded through the eligibility criteria. The search yielded 3675 records, all of which were exported to EndNote X9 (Clarivate, Philadelphia, PA, USA) for reference management and subsequent eligibility screening.

Studies were considered eligible if they directly addressed food waste at the consumer or household level, or within retail-consumer and food-service consumption settings, and provided substantive evidence on at least one of the following: behavioral drivers or decision-making processes, food management practices, or interventions intended to prevent or reduce food waste. Studies conducted in retail or food-service settings were included only when they examined consumer behavior or features of the consumption environment that influenced food waste generation or prevention.

**Figure 2:**
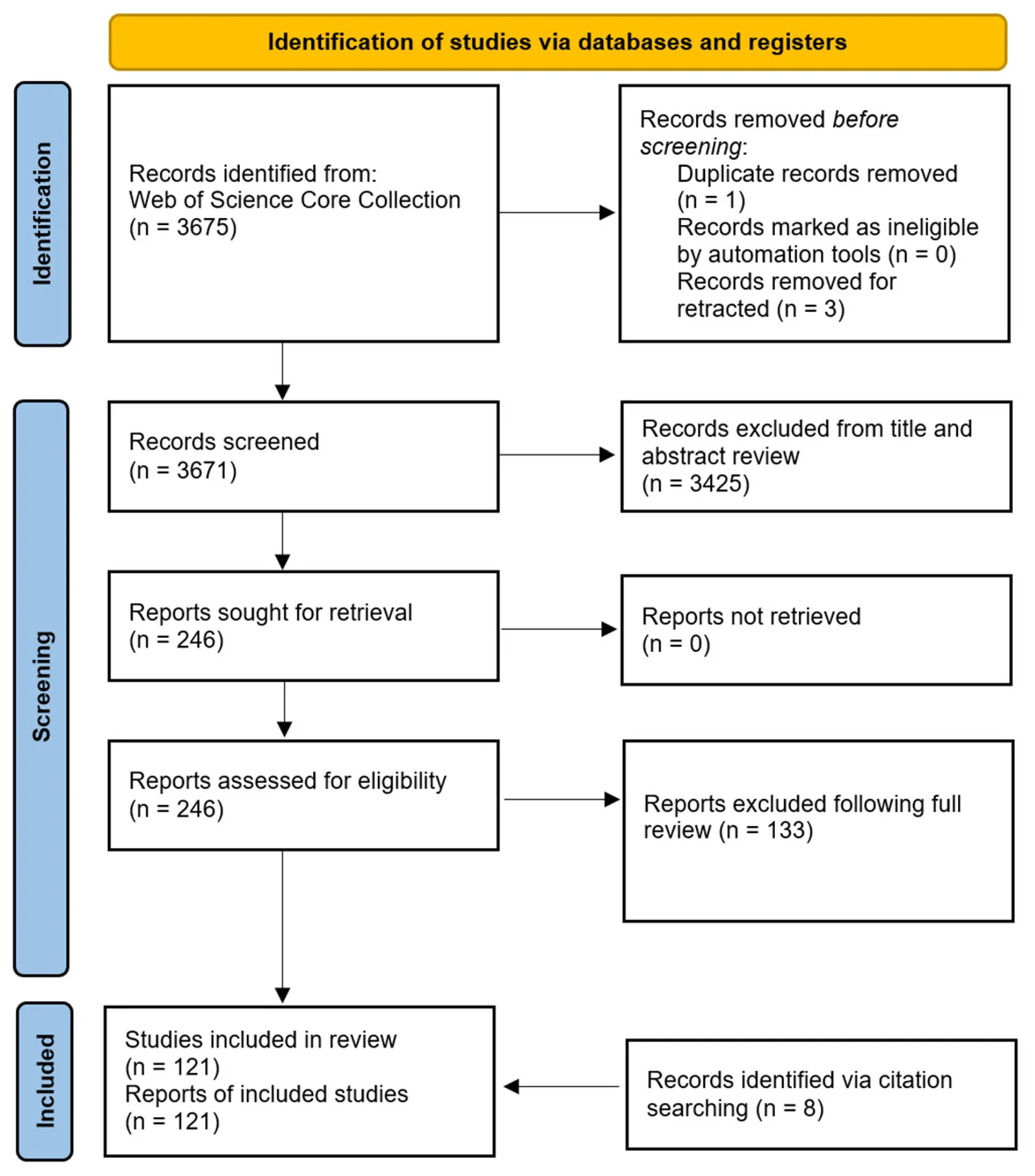
Flowchart of literature search and selection based on PRISMA 2020.

Following export to EndNote X9, the titles and abstracts of the retrieved records were screened against the scope and objectives of the review. Records were excluded when they did not directly examine consumer- or consumption-stage food waste, or when food waste was mentioned only incidentally and did not constitute a central outcome or analytical focus. Among the records excluded at this stage were studies primarily concerned with pre-consumption food loss, downstream waste treatment or valorization, general waste management, and other topics unrelated to consumer food waste behavior or prevention. Potentially eligible articles were subsequently assessed in full text against the inclusion criteria. At this stage, articles were excluded if they did not provide substantive evidence directly relevant to consumer food waste behavior, behavioral drivers, food management practices, or reduction interventions. This screening process yielded 113 studies from the structured database search. Backward and forward citation tracking of these studies was then conducted to identify additional relevant publications that may not have been captured by the original search strategy. Eight additional studies met the same eligibility criteria and were included, resulting in a final core corpus of 121 studies. Additional sources cited for conceptual definitions, background information, policy context, and the broader governance discussion were used as supplementary references and were not counted among the 121 included studies. These supplementary sources were not subject to the same eligibility and screening procedure as the core corpus, but were selected according to their relevance to the specific conceptual or contextual issues discussed and, where applicable, the authority and appropriateness of the sources. Figure 2 shows the selection process of the included studies. The screening process involved two authors. One author conducted the initial title and abstract screening, while both authors independently assessed the full texts of potentially eligible studies against the predefined inclusion criteria. Any differences in eligibility judgments were discussed until agreement was reached.

Two authors independently extracted information from the included studies. For the included studies, basic information was recorded on the author(s), year of publication, publication source, research setting, study design, and principal findings. Within this common framework, additional information was organized according to the study type and the thematic subsection to which the study contributed. For studies concerning behavioral drivers, particular attention was paid to the factors associated with consumer food waste and the corresponding findings. For studies of interventions to reduce food waste, particular attention was paid to the specific intervention measures, the principal reported outcomes, and relevant implementation conditions or limitations. Differences in extraction or interpretation were resolved through discussion and re-examination of the relevant full texts until consensus was reached. No formal methodological quality assessment was conducted. However, differences in study design, research setting, and outcome measurement were considered when interpreting the findings.

The thematic synthesis proceeded through two main analytical steps. First, studies on the behavioral drivers of consumer food waste were compared and grouped according to whether they primarily explained waste-related decisions and practices through cognitive judgments and perceptions, motivational priorities and goal conflicts, or environmental and situational conditions. This process led to three broad categories: cognitive biases, motivational drivers, and environmental and situational influences. Second, studies of interventions to reduce food waste were classified according to their primary behavioral logic, resulting in five intervention types: information-based interventions, social norm interventions, choice architecture and environmental design interventions, incentive-based interventions, and education and capacity-building interventions. The two authors jointly classified the included studies using the analytical criteria described above. Where a study contributed to more than one thematic category, its primary placement was determined through discussion, while relevant cross-category contributions were retained in the narrative synthesis. Differences in judgment were resolved through re-examination of the relevant full texts until consensus was reached.

**Figure 3:**
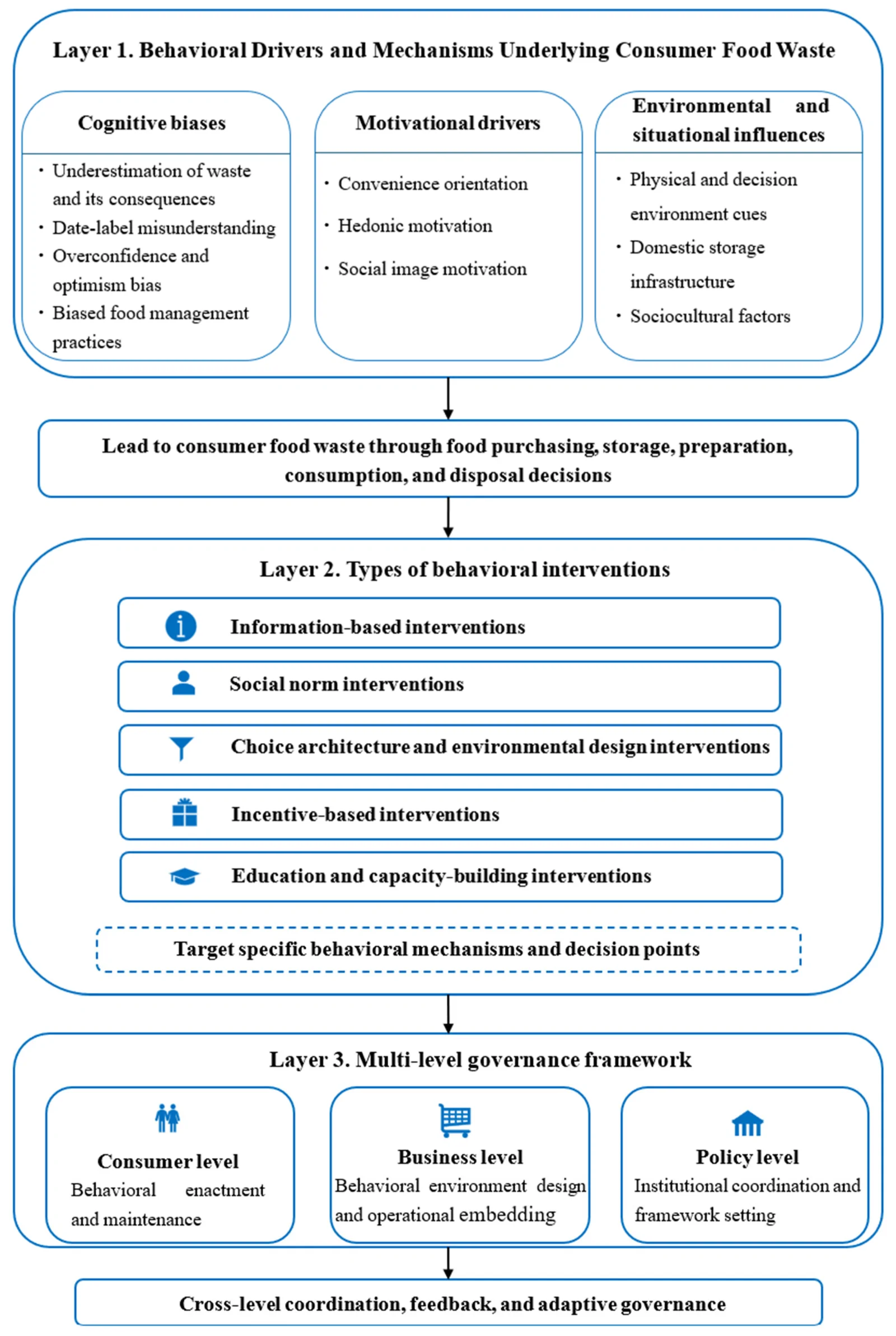
Analytical framework for consumer food waste reduction.

Building on these two steps, the review developed a multi-level governance perspective to interpret how behavioral interventions can be supported, implemented, and sustained through interactions among consumers, businesses, and policymakers. This perspective was not derived from a separate literature search focused specifically on governance, but was developed as an integrative analytical framework for connecting behavioral drivers, intervention types, and governance roles. Figure 3 presents the analytical framework of this review.

## 3. Behavioral Drivers and Mechanisms Underlying Consumer Food Waste

Understanding why consumers waste food is a necessary starting point for designing effective behavioral interventions and for clarifying where governance support is needed. Consumer food waste is not the result of a single irrational decision, nor can it be sufficiently explained by a simple lack of awareness or moral concern. Rather, it develops through a sequence of food-related decisions across purchasing, storage, preparation, consumption, reuse, and disposal. These decisions and practices are shaped by bounded rationality, competing goals, habitual routines, and immediate environmental cues [31].

Building on insights from psychology, behavioral economics, and consumer behavior research, this review organizes the behavioral drivers of consumer food waste into three categories, namely cognitive biases, motivational drivers, and environmental and situational influences. These categories are analytically distinct but may overlap and, in some circumstances, reinforce one another. For example, consumers may misjudge future food needs, prioritize convenience over careful planning, and make decisions under storage conditions, dining conventions, or service environments that increase the likelihood of food waste. Identifying these drivers provides an analytical foundation for the intervention typology developed in the next section and for the multi-level governance framework proposed later in the article.

### 3.1. Cognitive Biases

Cognitive bias refers to systematic deviations from fully rational judgment that arise when individuals rely on intuitive inferences or heuristic rules during information processing and decision-making [32]. In the context of food consumption, such biases influence consumers’ perceptions of food value, safety, availability, and future needs [16,33,34,35]. Therefore, food waste does not always stem from deliberate neglect; it can also result from recurring estimation and judgment biases under conditions of uncertainty. Consumers often face high levels of uncertainty and incomplete information when estimating future appetite, interpreting date labels, assessing food edibility, or managing household inventories. Under these circumstances, they may rely on heuristics that generate systematic errors, and the accumulation of these errors can result in unnecessary food discard [36,37,38,39].

Consumer food waste is associated with several recurring cognitive tendencies. Consumers may underestimate the amount and consequences of the food they waste, misunderstand date labels, display overconfidence or optimism bias, and make biased judgments in everyday food-management practices. These tendencies are analytically distinct but often overlap and reinforce one another in practice.

#### 3.1.1. Underestimation of Waste and Its Consequences

Related studies have shown that consumers generally underestimate the extent of their own food waste and its potential consequences; such systematic underestimation has been identified as an important cognitive barrier to waste-reduction behaviors [40]. It can weaken individuals’ sense of urgency and reduce the likelihood that they will view waste reduction as a meaningful consumption goal. It is not merely a lack of information, but a biased perception through which consumers regard the waste they generate as less frequent, less extensive, and less consequential than it actually is.

Consumers often underestimate the amount of food they discard, particularly in everyday household settings where waste accumulates gradually in small quantities rather than a single visible event [40,41,42]. They may also have a limited understanding of the environmental, social, and economic consequences of food waste, which weakens their motivation to reduce waste [43,44,45]. More broadly, consumers also have limited factual knowledge of the environmental impacts associated with food production and consumption [46], suggesting that the environmental costs embodied in discarded food may not be readily apparent. Consumers may also underestimate the cumulative contribution of routine food-management decisions to avoidable waste [47,48]. These cognitive gaps help explain why consumers who are generally concerned about sustainability may still engage in wasteful behaviors.

Existing studies suggest that correcting consumers’ underestimation of food waste and strengthening their sense of responsibility do not necessarily lead to behavioral change [49]. Although greater recognition of the seriousness of food waste is associated with lower levels of waste, this relationship is shaped by personal habits, practical skills, and situational constraints [50]. Awareness-raising alone is therefore unlikely to generate sustained behavioral change. It may be more effective when combined with concrete behavioral guidance and practical support that help consumers translate awareness into everyday waste-reduction practices.

#### 3.1.2. Date-Label Misunderstanding

Misunderstanding food date labels is a major cognitive driver of avoidable household food waste [51,52,53]. Date labels are intended to convey different types of information: “best before” dates primarily indicate the period during which food retains its expected quality, whereas “use by” dates indicate the period during which food can be safely consumed [54]. However, many consumers do not clearly distinguish between these terms. Consequently, consumers may treat quality-related date labels as fixed deadlines for disposal, even when the product may remain acceptable and sensory assessment would be appropriate [55,56,57].

This phenomenon is not merely caused by insufficient knowledge of food labels. Instead, it reflects consumers’ precautionary responses to uncertainty when judging whether food is still safe or acceptable to eat. When consumers are unsure about the edibility of a food item, they may adopt a precautionary discard decision, treating disposal before or shortly after the labelled date as the safer option, even when the food may still be edible [58]. This tendency is reinforced when labeling systems are fragmented, terminology varies across products and jurisdictions, and consumers lack confidence in using sensory cues to assess whether food can still be consumed [59,60,61,62].

Improving consumers’ factual understanding of label meanings may not, by itself, be sufficient to prevent premature disposal [37]. Even when consumers are able to distinguish between different labels conceptually, their actual decisions may still be shaped by food-related risk tolerance, loss aversion, perceived safety concerns, and product characteristics [63,64]. This suggests a gap between label understanding and disposal behavior. It also indicates that the behavioral effects of date labels depend not only on the clarity of information provided, but also on how consumers perceive and manage uncertainty, risk, and responsibility in everyday food practices. From an intervention perspective, standardizing label terminology is an important step, but it is unlikely to substantially reduce food waste unless accompanied by guidance that helps consumers distinguish quality deterioration from genuine safety risks and strengthens their confidence in making appropriate edibility judgments.

#### 3.1.3. Overconfidence and Optimism Bias

Overconfidence and optimism bias are important cognitive mechanisms contributing to consumer food waste. Overconfidence refers to excessive confidence in one’s own knowledge, judgments, or abilities, and may involve both overestimation of one’s performance and over-precision in one’s beliefs [65]. Optimism bias refers to the tendency to view one’s own outcomes or behavior more favorably than is objectively justified. In food consumption contexts, these biases can distort consumers’ assessments of their food-related knowledge, predictions of future preferences and consumption, and their evaluations of their own waste behavior.

In planning, purchasing, and household food management, consumers may assume that their current preferences and intentions will persist into the future [66]. They may show overconfidence in forecasting future food preferences and overestimate the extent to which current liking predicts future consumption, while underestimating the influence of fluctuating appetite, changing schedules, and situational cues on actual eating behavior [67]. These prediction errors may contribute to mismatches between the quantities selected or purchased and the amounts eventually consumed.

Overconfidence also affects how consumers interpret food-related information and make discard decisions. Consumers may feel confident in their ability to understand date labels, assess freshness, or judge whether food remains acceptable to eat, even when their actual knowledge is limited. Such knowledge overconfidence is associated with earlier date-related disposal and may reduce consumers’ receptiveness to corrective label information [33]. Optimism bias has likewise been identified as an important predictor of food-waste decisions, including the disposal of food that may still be edible and leftovers that could otherwise have been consumed [68]. Food-safety misperception and consumption misestimation have been identified as two important and potentially interacting pathways to consumer food waste [69].

These biases may be further amplified by situational constraints. Research on dietary decision-making shows that, under objective time pressure, overconfident consumers may experience higher subjective time pressure and greater perceived choice difficulty [70]. This suggests that when consumers make food-related judgments under time pressure, limited attention, or everyday demands, overconfidence may weaken their recognition of uncertainty and increase their reliance on personal experience and immediate judgment. In the context of food waste, overconfidence and optimism bias may increase the risk of premature disposal by reinforcing unrealistic consumption expectations, excessive reliance on personal food-safety judgments, and miscalibrated food-related judgments and expectations. These findings suggest that interventions should not be limited to providing information, but should also help consumers calibrate their confidence and expectations. By making the uncertainty of future consumption, the limits of personal food-safety judgments, and the consequences of overly optimistic assumptions more salient, such interventions may help reduce disposal decisions caused by insufficient information or hasty judgment.

#### 3.1.4. Biased Food Management Practices

Household food waste is closely related to biased judgments embedded in routine food-management decisions across planning, purchasing, storage, cooking, and leftover reuse. In these processes, consumers may inaccurately estimate household needs, fail to monitor existing food stocks, or make insufficient adjustments to changes in meal plans and consumption situations, which can gradually increase the likelihood of avoidable waste [36,47,71]. Although each judgment error may appear minor, repeated errors across different stages of food management can accumulate over time and eventually result in substantial waste. Thus, household food waste rarely arises from a single erroneous decision; it is more often produced through a sequence of interrelated judgments and routine habits within everyday food management.

During planning and purchasing, consumers may underestimate existing stocks, overestimate future needs, or overlook changes in household schedules, leading to insufficient meal planning, repeated purchases, and over-purchasing. These biased judgments and management shortcomings can intensify the mismatch between the amount of food acquired and the household’s actual consumption capacity, thereby increasing the likelihood that food will be left unused, spoiled, and discarded [72,73,74]. During storage, consumers may fail to monitor existing stocks accurately, prioritize foods that should be consumed first, or distinguish quality deterioration from genuine food-safety risks. These limitations can result in delayed use, premature disposal, or the accumulation of food that is unlikely to be consumed [75,76,77,78,79]. During cooking and leftover handling, limited cooking and food-management skills, including inadequate storage practices and difficulty repurposing leftovers into new meals, together with weak motivation to reuse remaining food and negative perceptions of leftovers, may reduce leftover reuse and increase the likelihood of disposal [80,81,82].

These practices have cumulative effects across successive stages, as early errors in planning and purchasing can create later difficulties in storage, preparation, and leftover reuse. Excess food generated by overestimating future needs or failing to check existing stocks may remain unused until its quality deteriorates, making disposal appear increasingly difficult to avoid and obscuring its connection with earlier preventable management shortcomings. Household food waste should therefore be understood as the outcome of a continuous food management process rather than solely as a final disposal decision. Interventions should provide practical support for meal planning, inventory checking, demand and portion estimation, freshness assessment, cooking skills, and leftover reuse, thereby strengthening everyday food-management capabilities and reducing the accumulation of errors across successive stages.

### 3.2. Motivational Drivers

Beyond biases in judgment and information processing, consumer food waste behavior is also shaped by motivational drivers. Food-related decisions often involve trade-offs among multiple goals [83], such as saving time and money [84], seeking enjoyment [85], showing care for others [83], maintaining social image [86], or reducing waste [87]. When these goals conflict, wasteful behavior becomes more likely if consumers prioritize convenience, hedonic gratification, or social approval over waste reduction. Food waste may therefore reflect a goal conflict in which waste reduction is accepted in principle but given lower priority in practice, rather than indifference to sustainability.

Convenience orientation, hedonic motivation, and social image motivation represent three important motivational drivers that may increase food waste risk [86,88,89]. Although these motivations can be distinguished analytically, they often overlap and reinforce each other in real-world decisions. For example, consumers may purchase quantities beyond their immediate needs because doing so feels convenient, pleasurable, and socially desirable.

#### 3.2.1. Convenience Orientation

Convenience orientation generally refers to a relatively stable tendency to minimize the time, physical effort, and mental effort involved in food-related practices, especially meal preparation, food handling and consumption [90,91]. In contemporary food environments, convenience is not merely a product or service attribute, but an important behavioral logic shaping everyday food-related decisions. Convenience orientation may contribute to food waste not simply because consumers prefer speed or ease, but because waste prevention depends on a series of everyday food management practices that require sustained attention, skills, and effort. Consumers with a stronger convenience orientation may therefore be more likely to rely on low-effort food practices and less willing to engage in careful food management, thereby increasing the likelihood of avoidable waste [88,92].

Household and shopping studies show how this mechanism operates in everyday consumption. Convenience-oriented consumers tend to report more food waste incidents and show lower willingness to choose discounted suboptimal foods, especially when such foods require additional handling or timely use [88]. Similar patterns are observed among consumer segments that rely heavily on convenience food. Casual consumers and kitchen evaders report higher levels of waste, but for different reasons: the former are more closely associated with “buy a lot and waste a lot” patterns, whereas the latter are characterized by low cooking involvement and limited advance planning [93]. Convenience therefore affects not only food acquisition, but also consumers’ willingness to engage in post-purchase food management. When food no longer fits immediate consumption needs, disposal may be perceived as a comparatively low-effort response.

Food delivery apps further intensify this convenience logic. By reducing ordering effort, expanding food choices, and enabling immediate delivery, food delivery platforms may encourage consumers to order more food than needed. Over-ordering is shaped not only by consumers’ attitudes, perceived convenience, trust, and expectations about leftover reuse, but also by platform arrangements and consumption conditions, including minimum-order thresholds, bundled promotions, fixed or unclear portion sizes, and limited facilities for storing leftovers [94,95,96,97]. Platform-mediated convenience therefore extends the waste mechanism from individual food management to the interaction among consumer motives, app design, platform arrangements, and post-purchase handling conditions.

Across these contexts, convenience shapes food waste through multiple pathways. It may increase waste risk by reducing the effort invested in food management, making it harder to match food acquisition or ordering with actual needs, and increasing the likelihood of over-acquisition, over-ordering, or quantity mismatches. This also suggests that interventions targeting convenience-oriented consumers should not rely only on additional consumer attention or self-regulation. Instead, waste prevention needs to be made more convenient itself, for example through clearer portion and quantity information, more flexible portion and bundle design, and easier support for storage, reheating, and leftover reuse.

#### 3.2.2. Hedonic Motivation

Hedonic motivations refer to individuals’ pursuit of pleasure, sensory gratification, positive emotional experience, and enjoyment in consumption. Within individual-level value theory, hedonism is conceptualized as a basic value type centered on pleasure and self-gratification [98]. At the cultural level, a related emphasis on pleasure, excitement, and variety is reflected in the value orientation of affective autonomy [99]. In food consumption contexts, such motivations are especially salient because eating satisfies physiological needs while also providing enjoyment, relaxation, and emotional reward. When consumers associate a satisfactory eating experience with novelty, variety, freshness, or immediate enjoyment, hedonic motivations may increase food waste risk.

Hedonic motivations do not necessarily lead directly to food waste. Their influence is more likely to be indirect, context-dependent, and specific to particular stages of food consumption and management. They may weaken certain waste-preventing practices, encourage the selection or acquisition of food beyond immediate needs, and shape sensory judgments about whether food remains desirable to consume. Hedonic motivations have been found to show relatively weak and stage-specific associations with household food waste prevention, with their effects becoming most visible in leftover management [85]. Higher hedonic shopping value has also been found to be negatively associated with food waste prevention behavior [89]. In travel contexts, consumers’ pro-environmental behavior may weaken as novelty seeking and letting oneself go increase the relative importance of hedonic and gain goals in food consumption decisions [100]. Hedonic considerations may also influence the perceived desirability of remaining food. Leftovers, for example, may become unwanted when they are perceived as providing a less satisfactory sensory experience than freshly prepared food, even when they remain edible [101].

Hedonic motivations are more likely to increase food waste risk when experience-oriented consumption takes priority over waste prevention. Pleasure-seeking itself is not the central issue; waste risk arises when preferences for freshness, variety, sensory appeal, and immediate satisfaction lead to over-selection or reduce the perceived desirability of remaining food. This reasoning suggests that interventions should not rely exclusively on moral appeals or messages of restraint. Instead, waste prevention may be more effective when it is made compatible with consumer satisfaction, perceived food quality, and pleasurable eating experiences.

#### 3.2.3. Social Image Motivation

Food consumption satisfies physiological needs while also serving as a socially meaningful form of self-presentation [86]. When ordering, eating, and dealing with leftovers, consumers may consider not only their actual needs but also how they are perceived by others, including whether they appear appropriate, generous, well-prepared, or socially respectable. Food waste may therefore reflect choices made in specific interactional contexts to support impression-management and identity-related goals, rather than simply negligence or poor food management [102,103,104].

Related studies point to at least two prominent pathways through which social image motivation may increase food waste risk. In social dining contexts, consumers may order excessively to avoid appearing stingy, underprepared, or insufficiently hospitable, thereby using food as a symbolic resource for displaying warmth, generosity, and social propriety. Ordering with companions can heighten impression-management motivation and over-ordering tendencies [103]. Mianzi may also weaken the constraining influence of personal norms on over-ordering attitudes [105], while face-saving concerns have been associated with lower intentions to pack leftovers [106]. Within online and social media environments, food can serve as a signal of lifestyle, taste, and social position. Food images, influencer-promoted restaurants, and the display-oriented consumption of others may stimulate conformity, social identity, and approval-seeking tendencies, encouraging symbolic, conspicuous, or impulsive food choices that depart from actual consumption needs and thereby increase the risk of over-ordering and subsequent waste [104].

Social image motivation therefore highlights a form of goal conflict shaped by social evaluation. When resource conservation conflicts with the maintenance of a favorable personal image, particularly in situations where social evaluation is public and immediate, consumers may give greater priority to image-related goals. However, social image motivation does not necessarily increase waste; its behavioral consequences depend on which forms of consumption are socially recognized and encouraged. Accordingly, in social settings, relying solely on knowledge dissemination or information provision is often insufficient to effectively reduce food waste. More effective interventions should combine practical information, social norm guidance, and image-based framing, so that moderate ordering, leftover packing, and waste reduction are perceived as respectable, responsible, and socially approved behaviors that are linked to a positive personal image.

Overall, motivational drivers influence food waste by shaping which goals consumers prioritize when waste reduction conflicts with convenience, enjoyment, or social approval. These drivers do not inevitably lead to waste, but their effects may be activated, amplified, or constrained by the physical and social environments in which food-related decisions take place.

### 3.3. Environmental and Situational Influences

Beyond individual cognitive and motivational processes, consumer food waste is also strongly shaped by the environments in which food-related decisions occur. Food choices are not always based on sustained rational deliberation but are often influenced by non-deliberative processes, habitual scripts, and situational cues [31,107]. Features of physical settings, household infrastructures, and sociocultural contexts can shape consumers’ judgments of what is normal, appropriate, sufficient, or desirable. Food waste may therefore occur even without an explicit intention to waste [77,108,109]. Rather than reflecting individual preferences or cognitive limitations alone, it is also conditioned by the material arrangements and social expectations embedded in consumption environments.

This section organizes environmental and situational influences into three broad dimensions: physical and decision environment cues, domestic storage infrastructure, and sociocultural factors. These influences operate across different scales, ranging from the micro-design of tableware and menus, to household storage facilities, to broader cultural expectations surrounding abundance, hospitality, and shared meals. Together, they help explain why food waste persists across diverse contexts, even when consumers express positive attitudes toward waste reduction.

#### 3.3.1. Physical and Decision Environment Cues

Micro-level design features within consumption settings can influence food-related decisions when consumers do not engage in extensive deliberation. Tableware, food presentation, serving formats, menu structures, and promotional rules together constitute contextual cues within the physical and decision environment. By shaping consumers’ judgments of appropriate portion sizes, food availability, consumption value, and purchase quantities, these cues can alter their food selection, ordering, and purchasing behavior, potentially increasing the risk of food being left uneaten and subsequently discarded.

Visual and spatial cues can influence consumers’ judgments of food portions. Plate size may affect portion perception through size-contrast effects, making the same amount of food appear smaller on a larger plate and thereby increasing the likelihood of excessive self-serving [110,111]. A field study conducted in a university dining hall found that replacing larger round plates with smaller oval platters was associated with reductions in both the amount of food selected and plate waste [112]. In foodservice settings, self-service formats, the variety of dishes available, the continuous availability of food, and specific serving arrangements can influence consumers’ judgments of appropriate serving quantities and consumption value. The pursuit of value for money, the desire to sample a wider variety of dishes, and operational practices intended to maintain an appearance of abundance may contribute to excessive self-serving and food leftovers in buffets and other large-scale dining settings [113,114]. In addition, different combinations of serving formats, moral appeals, rewards, and penalties can affect the amount of plate waste generated [115]. In retail settings, multi-unit promotions and other promotional designs that encourage larger purchase quantities may lead consumers to buy more food, leaving some perishable items unconsumed and thereby increasing household food waste [116]. In digital food-ordering environments, the presentation of portion options and the clarity of portion-size information can shape the quantities ordered and, in turn, the likelihood of subsequent food waste [117,118].

Food waste interventions should not rely solely on consumers’ conscious management after food has been purchased or served. Adjusting plate size, providing more appropriate portion options, improving serving processes and food presentation, and redesigning promotional rules that encourage excessive purchasing can intervene earlier in the formation of serving and purchasing decisions, thereby reducing reliance on consumers’ sustained attention and self-control. However, the effectiveness of such environmental designs remains dependent on the specific consumption setting, the way in which they are implemented, and their coordination with other intervention measures.

#### 3.3.2. Domestic Storage Infrastructure

Household food waste is also shaped by domestic storage infrastructures and material arrangements. Refrigerators, freezers, cupboards, containers, packaging, and available kitchen space influence where food is placed, how visible it remains, and whether it can be easily identified and used. Food stored out of sight or in poorly organized spaces may be overlooked until its quality deteriorates [119,120]. Domestic storage environments therefore affect waste by increasing or reducing the effort required to monitor and rotate household food stocks.

Storage conditions influence the physical preservation of food. Refrigerator performance, temperature and humidity, compartment design, packaging functionality, and food-specific storage requirements affect freshness, shelf life, spoilage risk and the practical usability of stored food [77,78,121,122]. Inadequate temperature control may accelerate deterioration, while unsuitable packaging may shorten usable life, be difficult to empty, or come in quantities that are poorly matched to household needs. Appropriate conditions can provide more time for consumption. Prolonged storage and perceived food suboptimality are also commonly reported reasons for household food disposal, particularly in incidents involving commonly wasted categories such as fresh produce and leftovers [123]. Improved storage conditions, however, do not necessarily reduce food waste under all circumstances. Although appropriate refrigeration can extend shelf life [78,122], evidence from rural China indicates that refrigerator use may also increase the amount of food stored at home and raise waste risk when additional stocks are not consumed in time [124]. Storage infrastructure has an ambivalent role; refrigeration can improve preservation, but it may also facilitate food accumulation.

This perspective broadens explanations of household food waste beyond individual awareness and decision-making by highlighting the interaction between domestic infrastructures, material environments, and everyday practices. Interventions should improve the practical conditions of food storage through clearer guidance, better-organized storage arrangements, and packaging designs that facilitate food identification and use. Such measures can make stored food easier to monitor and consume before its quality deteriorates.

#### 3.3.3. Sociocultural Factors

Beyond immediate material settings, food waste is embedded in broader sociocultural systems. Food provisioning, sharing, and leftover disposal are shaped by cultural values, social norms, and established practices that define the meanings of abundance, thrift, hospitality, care, and leftovers. In many contexts, preparing or serving more food than is actually needed may signify generosity, care, prosperity, or respect for others rather than being regarded simply as wasteful [109,125]. Overprovisioning may consequently become normalized or socially acceptable in particular settings and, in some cultural contexts, weaken the influence of personal norms against waste [126,127].

These influences are often reproduced through shared role expectations and situational conventions. Within households, culturally defined expectations surrounding the roles of mothers, caregivers, and food providers may encourage the purchase and preparation of more food than is immediately required, because maintaining ample supplies can symbolize care, responsibility, and household security [127,128]. Similar expectations may operate during family gatherings, festive meals, and hospitality occasions, where hosts are expected to provide sufficient variety and quantity and to avoid any appearance of shortage [109,125,129]. In these settings, surplus food may arise not only from inaccurate planning or weak food-management practices, but also from socially established standards of appropriate provision.

Sociocultural influences are not uniform, however. Norms of thrift, religious responsibility, and respect for food may discourage disposal or support leftover management in some practices and contexts, whereas norms emphasizing abundance, hospitality, and generous provision may increase the likelihood of surplus. The relative importance of these norms, as well as the acceptability of serving smaller quantities, reusing leftovers, or taking unfinished food away, varies across cultural and consumption contexts [127,129,130].

Food waste may therefore be sustained through shared practices and expectations even when individuals personally support waste reduction. Interventions should consequently address not only individual awareness and food-management skills but also the social meanings attached to adequate provision and leftover use. This may involve promoting moderate provision, flexible portions, and leftover reuse as culturally acceptable expressions of care, responsibility, and hospitality.

Consumer food waste is shaped by the combined influence of cognitive biases, motivational priorities, and environmental and situational conditions. Consumers may misjudge their food needs, prioritize convenience, immediate gratification, or social approval, and make decisions within environments that normalize abundance or make careful food management difficult. Food waste is therefore better understood as a multidimensional behavioral outcome rather than solely as the consequence of isolated individual choices. This understanding underscores the importance of designing interventions that are sensitive to these behavioral drivers and the contexts in which they operate. Table 1 summarizes these drivers and their intervention implications. The next section examines how different types of behavioral interventions seek to address these drivers of food waste.

## 4. Types of Behavioral Interventions

Behavioral interventions seek to reduce consumer food waste by changing the conditions under which everyday food decisions are made. They do so not only by increasing awareness, but also by reshaping information environments, social norms and expectations, choice structures, perceived costs and benefits, and practical capacities for action. From a multi-level governance perspective, these interventions should not be understood merely as consumer-facing tools. Their design and effectiveness often depend on business practices, such as digital interface design, foodservice routines, and product labeling systems, as well as broader policy support. This section therefore classifies behavioral interventions according to their primary behavioral logic while noting the governance conditions that shape their implementation and effectiveness.

### 4.1. Information-Based Interventions

Information-based interventions seek to reduce food waste by improving the quality, salience, and usability of the information available to consumers when food-related decisions are made. Food waste may be partly driven by information that is incomplete, unclear, difficult to interpret, overly abstract, or provided too late to guide action. These information problems may concern food safety and edibility, date labels, portion sizes, appropriate ordering, storage practices, or the consequences of disposal. Making such information more visible and understandable can correct misunderstandings, reduce uncertainty in food assessment, draw attention to waste at relevant moments, and help consumers identify practical ways to avoid it. Depending on the message and the setting, information may also affect perceived health risks, personal norms, and consumers’ tendency to rationalize wasteful choices.

Date-label research shows both the value and the limitations of this approach. Explaining the meaning of date labels can improve consumer understanding and reduce intentions to discard food prematurely under uncertainty [14]. Information about the edibility of food beyond the best-before date may also lower perceived health risks and strengthen personal norms against unnecessary disposal, although these effects vary across behavioral and social contexts [131]. Simply increasing the visibility of information, however, does not guarantee behavioral change. Consumers preferred the addition of Look–Smell–Taste symbols on food products, but these symbols did not significantly increase their acceptance of food beyond the best-before date, their use of sensory assessment, or their actual use of the products [132]. The usefulness of label information therefore depends not only on whether it is noticed, but also on whether consumers can interpret it confidently and incorporate it into established food-assessment practices.

Similar variation is evident in restaurant and household settings. Menu, table, and buffet prompts [15,133,134,135], waste-reduction messages [136,137], portion-size information and menu-based decision support [138], and household storage prompts [13] all seek to make relevant information available close to the decision it is intended to influence. A table prompt available during the meal, for example, can encourage customers with leftovers to request a takeaway container when the prompt identifies a clear and easy action [15]. Before ordering, menu designs that display portions alongside familiar reference objects may help consumers estimate quantities more accurately and reduce simulated over-ordering by narrowing the information gap between restaurants and diners [138]. In the home, marking a designated “use-it-up” area or labeling food that should be consumed first can increase the visibility of stored products and support meal planning and communication among household members [13].

The way information is framed also matters. In a Chinese hotpot restaurant, instructive information offering guidance on appropriate ordering and consumption significantly reduced directly measured plate waste, whereas guilt-based and combined messages did not significantly reduce total waste [137]. Concrete prompts such as reminders to order moderately or take leftovers away were likewise more effective than broad appeals such as “cherish food,” partly because they left consumers less room to justify wasteful choices [133]. This does not mean that greater message concreteness will always produce stronger effects. The advantage of concrete over abstract prompts may weaken among consumers who perceive explicit behavioral guidance as a threat to their autonomy, suggesting that clarity should be balanced with an appropriate tone and sensitivity to audience characteristics.

Evidence varies across both intervention types and outcome measures. Some field studies report reductions in directly measured plate waste, including information nudges at a hotel breakfast buffet [135], and instructive messages in a restaurant [137]. A household storage prompt was also associated with lower self-reported food waste, although the absence of a control group limits causal interpretation [13]. Other interventions mainly affected intentions or self-reported behavior. Environmental messages increased waste-reduction and meal-planning intentions [136], while a salience prompt reduced self-reported restaurant leftovers. Adding portion-size information and hunger-related task knowledge did not produce a stronger effect, and neither informational condition produced a detectable change in aggregate weighed waste [134]. These differences highlight the need to distinguish between cognitive outcomes, intentions, self-reported behavior, and objectively measured food waste.

Information-based interventions are therefore more likely to be useful when they reduce uncertainty, make waste salient, and lower the effort required to identify an appropriate action at the moment of decision. They are low-cost, scalable, and easy to deploy, but evidence that their effects persist after the information is removed is still limited. Their effects may be strengthened when clear communication is accompanied by feasible choices, practical capabilities, and supportive organizational arrangements. This also places their implementation within a wider governance structure; policy frameworks can establish consistent standards for consumer-facing information and labeling, businesses can translate these standards into clear and reliable communication, and practical guidance can be embedded in the points at which consumers purchase, order, store, consume, and dispose of food.

### 4.2. Social Norm Interventions

Social norm interventions seek to reduce consumer food waste by reshaping perceptions of what others commonly do and which behaviors are socially approved. Whereas information-based interventions improve the quality, salience, and usability of the information available for food-related decisions, social norm interventions influence how consumers evaluate their own conduct in relation to relevant others and perceived social expectations. Food waste, from this perspective, is not simply a private outcome of individual choice. It is embedded in social relations and cultural expectations that influence whether abundance, restraint, leftover use, and waste prevention are regarded as normal or appropriate. Social norm interventions are therefore closely connected to motivational and sociocultural influences such as conformity, image maintenance, and the meanings attached to hospitality and adequate provision. The theoretical basis of this approach rests on the distinction between descriptive and injunctive norms. Descriptive norms provide information about what others do, whereas injunctive norms indicate what behaviors are socially approved or expected [139,140]. Norms are especially influential when they are salient in the immediate decision context [139].

Descriptive and injunctive norms operate through different pathways. Descriptive norms provide a social reference for what is common in a setting, while injunctive norms convey whether a behavior is considered appropriate and may operate through anticipated approval or disapproval. Cross-cultural experimental evidence shows that both types of message can strengthen food waste prevention intentions by changing perceived norms, although the strength of these effects varies across cultural contexts [140]. Subjective norms may also influence intentions indirectly by contributing to personal moral obligations, thereby linking perceived social expectations with a sense of responsibility for reducing waste [141]. Thus, social norm interventions work by activating perceptions of what is common, socially approved, and considered appropriate.

Applied studies show that social norm interventions can influence waste-related behavior, although their mechanisms are not always easy to isolate. In restaurants, table-tent messages have encouraged diners to take leftovers home and reduced food discarded on site, but their effects may reflect descriptive norm information, direct prompts, reminders, and signals that takeaway is socially acceptable [142]. In corporate dining, a combined intervention linking social norm messages with portion-prompting signage reduced objectively measured plate waste, showing that normative appeals can form part of an effective point-of-decision intervention [143]. Household studies suggest that social norm messages can complement practical support. Food-management packages containing measuring tools, labels, recipes, and other guidance can improve waste-prevention practices, while norm elements may strengthen some behavioral changes without necessarily producing an additional reduction in reported waste [144]. More direct evidence of complementarity comes from a field study in which the clearest reduction in household grain waste occurred when a descriptive norm message was combined with a measuring cup that supported accurate portioning [145]. Norm effects also depend on message design. Descriptive norms appear more compatible with positive framing, whereas injunctive norms may be better aligned with negative framing, and these relationships vary across dining contexts [146].

Social norms should not be treated as uniformly beneficial. Messages emphasizing the prevalence of wasteful behavior may unintentionally make such behavior appear normal [139]. Existing expectations surrounding abundance, hospitality, and social image can also make overprovisioning or leaving food appear acceptable or socially appropriate in particular settings [109,125,126]. Delivery channels matter as well. A retailer-led Facebook campaign did not produce greater reductions in self-reported household food waste than information-based communication or the control condition, indicating that socially oriented online content does not necessarily generate additional reductions [147]. Social media may still help diffuse norms, identity cues, social support, and feedback, particularly in tourism and hospitality, but these approaches require clearer behavioral targeting and stronger evaluation [148].

Social norm interventions are more likely to be effective when they identify a relevant reference group, communicate a credible and culturally appropriate expectation, and connect that expectation to a specific action consumers can realistically perform. General moral appeals are unlikely to be sufficient when consumers lack clear guidance, practical tools, or supportive arrangements. Restaurants, retailers, and digital platforms can reinforce waste-reducing norms by making appropriate portions, leftover takeaway, and other preventive actions more visible and socially acceptable. Public communication and policy frameworks may also help shape broader meanings of responsible consumption. Social norms are therefore shaped not only through interpersonal influence but also through business practices, institutional settings, and public communication.

### 4.3. Choice Architecture and Environmental Design Interventions

Choice architecture and environmental design interventions seek to reduce food waste by restructuring the immediate context in which food-related decisions are made. Rather than relying primarily on information, persuasion, or sustained self-regulation, these interventions alter the arrangement of options, defaults, physical cues, portion formats, and service processes to make lower-waste choices easier or more salient at the moment of action [149]. By modifying the physical and procedural features of immediate food-choice environments, they can influence behavior while reducing the cognitive effort required to make less wasteful choices.

Physical cues and portion conditions shape how much food consumers initially select. In a university dining hall, replacing larger round plates with smaller oval platters reduced initial food selection and plate waste [112]. In a worksite cafeteria and an upscale restaurant, offering reduced-size entrées alongside regular-size options was also associated with significantly lower plate waste [150]. Similarly, reducing the standard portion of French fries in an on-campus restaurant lowered consumers’ intake of French fries and plate waste [151]. These effects nevertheless vary with serving conditions. Smaller food units may reduce consumption but increase waste when consumers receive a fixed amount of food. When consumers served themselves, smaller units led them to select and consume less, while waste remained low and did not differ significantly by unit size [152]. Portion-based strategies are therefore more likely to prevent waste when consumers can adjust quantities before surplus is created.

Menu design can also influence the correspondence between ordered and consumed quantities by changing which portion options are most visible or effortless to select. A narrow menu displaying only a small portion, while still allowing consumers to request larger portions, reduced food orders and food waste without significantly lowering food intake [118]. The results are consistent with anchoring and menu-dependent self-control mechanisms, particularly among consumers facing a self-control dilemma at the point of ordering. Menu structure may therefore reduce over-ordering by making moderate portions more salient and requiring greater deliberation to select larger quantities.

The potential and limitations of environmental design are also evident in service processes. Trayless dining seeks to discourage over-selection by making it less convenient to carry multiple food and beverage items. One study reported a significant reduction in solid plate waste after tray removal [153], whereas another found lower food selection and consumption without a statistically significant reduction in food waste [154]. Changing the sequence in which food is served similarly attempts to influence consumption before waste occurs, but the results appear highly context dependent. In a large cluster randomized trial conducted in school canteens, serving vegetable side dishes before other meal components produced heterogeneous effects across school clusters and no significant effect for the full sample [155]. The effectiveness of service-process changes therefore depends on institutional arrangements, food characteristics, established eating practices, and implementation conditions.

Defaults applied to leftovers operate at a later stage because they intervene after surplus food has already been created. Providing a doggy bag by default can increase uptake by removing the need for consumers to request one actively and by mitigating the shame associated with doing so [156]. The outcome may nevertheless depend on how the option is presented, since opt-out arrangements can also affect perceived autonomy and evaluations of the restaurant and its service staff. A natural field experiment in Italian restaurants found that default provision did not significantly increase doggy-bag uptake, whereas a descriptive social-norm message did [157]. Default-based approaches may therefore interact with shame, social visibility, service expectations, and staff behavior. Because they primarily facilitate the recovery of leftovers rather than prevent over-ordering or over-serving, increased doggy-bag uptake should not be treated as evidence of waste reduction unless the subsequent consumption or disposal of the food is also assessed.

Several studies report favorable effects for interventions that shape food selection before surplus is created, including plate and portion design, self-service arrangements, and menu-based portion architecture [112,150,151,152]. Their effects, however, are not automatic. Design changes are more likely to reduce waste when they address a clearly identified behavioral mechanism and fit the service setting, cultural context, consumer expectations, and operational routines. Choice architecture thus shifts attention from individual discipline to the environments in which food decisions are structured, highlighting the role of restaurants, foodservice providers, and school canteen operators in designing immediate decision environments. Successful implementation also requires avoiding excessive inconvenience to consumers, diminished consumer satisfaction, and conflicts with established eating practices.

Collectively, information-based, social norm, and choice architecture interventions show that consumer food waste is shaped by what people know, what they perceive others expect, and how immediate choices are organized. Raising awareness alone is therefore unlikely to be sufficient when social expectations and decision environments continue to encourage excessive selection or disposal. Because the effects of these approaches remain sensitive to setting and implementation conditions, the following sections examine how incentive mechanisms and capacity-building measures may provide additional support.

### 4.4. Incentive-Based Interventions

Incentive-based interventions reduce food waste by adjusting consumers’ perceptions of the costs and benefits associated with food-related decisions. The negative consequences of over-purchasing, over-serving, or discarding food are often not immediately apparent, whereas the perceived benefits of obtaining or providing more food are more readily felt. By increasing the costs of wasteful behavior or offering rewards for waste reduction, incentives can make these consequences more concrete and salient to consumers, thereby encouraging more restrained food-related decisions.

Such interventions can operate at different stages of purchasing, serving, consumption, and disposal. Unit-based charging at the household or municipal level links disposal costs more directly to household behavior. An RFID-based weight-charging system, for example, produced a greater reduction in recorded food-waste disposal than a community-based charging arrangement in which costs were shared among households [158]. In foodservice settings, threatened penalties, clean-plate rewards, and discount-based schemes have been associated with reductions in observed plate waste, although their effects differ according to incentive design and service context [115,159,160]. Incentives can also be introduced before waste occurs. Discounts for removing unwanted side dishes and additional charges for extra items encouraged participants to select fewer side dishes in an experimental ordering context [161]. In retail settings, price discounts may increase consumers’ willingness to choose suboptimal foods, but the required reduction varies according to defect type and severity, accompanying product attributes, and consumer segment [162].

Whether these interventions are effective depends on how clearly the economic consequences are connected to behavior and whether consumers have practical opportunities to adjust their choices. A non-randomized comparison of Brazilian restaurant configurations found lower plate waste in pay-by-weight buffets, where both pricing and consumer control over food and portion selection differed from fixed-price table-service settings [163]. Financial consequences alone, however, do not necessarily produce the intended response. Large-scale field evidence from Chinese foodservice settings found no significant overall reduction from financial incentives implemented alone, while one tested combination of instructive information and a high-intensity reward reduced food waste [164]. Incentives are therefore more likely to influence behavior when they are accompanied by clear guidance and service arrangements that enable consumers to respond without excessive effort or loss of choice.

Responses also differ across consumer groups because of variations in consumer characteristics, price sensitivity, and reactions to particular intervention designs [159,162,164]. A reduction in visible plate waste does not necessarily indicate that the earlier decisions generating excess food have changed. A clean-plate reward increased the likelihood that consumers finished the food they had taken but did not reduce the amount initially selected, indicating that lower plate waste may result from greater consumption rather than more restrained selection [159]. Poorly designed or inappropriately calibrated incentives may also generate unintended consequences, including consumer resistance and weakened customer loyalty [115,160]. Penalty-based or overly strong financial incentives may be counterproductive when perceived as coercive or incompatible with the dining experience consumers seek [115,164].

Incentive-based interventions connect consumer behavior with institutional regulation and market-based policy instruments. Governments and municipalities can shape behavioral incentives through waste disposal charges and other economic measures, while businesses can translate these signals into pricing arrangements, portion options, ordering procedures, and reward mechanisms suited to retail and foodservice operations. Consumers must nevertheless be provided with feasible alternatives and sufficient control to respond without excessive effort or disruption to the consumption experience [161,163]. Monetary rewards and penalties may also be supplemented by digital badges, points, behavioral feedback, identity signaling, and gamified participation, which can strengthen engagement and social recognition when matched to the target behavior and integrated into the immediate service environment [148]. More durable waste-reduction outcomes may therefore be supported when policy establishes enabling rules and economic signals, businesses incorporate workable incentives into routine operations, and consumers can respond through everyday food-related decisions.

### 4.5. Education and Capacity-Building Interventions

Education and capacity-building interventions seek to reduce food waste by strengthening consumers’ practical competence and everyday food-management routines. Unlike information-based prompts that provide guidance at specific decision points, these interventions aim to develop knowledge, skills, confidence, and habitual practices over time. They are particularly relevant to limitations in everyday food management, including inadequate meal planning, disorganized storage, over-preparation, and difficulties in using leftovers. Household food waste is closely associated with routine practices across shopping, storage, meal preparation, and leftover management. Field experimental evidence also indicates that education focused on meal-planning skills can reduce diary-recorded domestic food waste, with an indirect pathway through improvements in consumers’ perceived meal-planning ability [47].

Consumers may support waste reduction in principle but still lack the practical means to act consistently on that intention. Education and capacity-building can address this gap by translating general waste-reduction goals into concrete practices, such as checking household stocks, preparing shopping lists, planning meals, storing and freezing food appropriately, interpreting date labels, adjusting preparation quantities, and reusing leftovers. Actionable guidance may be especially useful when combined with techniques that support implementation. An intervention integrating tailored action knowledge, goal setting, and public commitment improved participants’ perceived ability to prevent food waste and produced greater improvements in self-reported waste-prevention practices than those observed in the control group [165]. This supports the value of combining practical knowledge with support for putting that knowledge into action.

Evidence from household interventions indicates that these approaches can improve specific food management practices and, in some cases, reduce measured waste. Multimedia programs addressing shopping, storage, date labels, freezer use, and leftover management have been associated with lower household food waste and improvements in behavioral conditions related to waste prevention [166]. Skills-based programs have also improved planning, freezing, leftover use, and date-label-related practices, together with consumers’ confidence in managing food [167]. Evidence of actual waste reduction is less consistent. Some studies report declines in measured waste, whereas others find improvements in practices and self-efficacy without statistically significant reductions [166,167]. Whether practical knowledge results in less waste may therefore depend on participant engagement, the relevance of the intervention to household circumstances, and the extent to which newly acquired practices are sustained.

These skills can also be reinforced through shared household routines and community-based activities. Programs combining cooking activities, meal-planning and storage resources, household toolkits, and reminders have been feasible and well received and have shown some potential to reduce avoidable fruit and vegetable waste [168]. A recent intervention protocol placed greater emphasis on food agency and on the combined role of motivation, opportunity, and ability, recognizing that consumers need both practical competence and suitable conditions for action [169]. Community-based evidence similarly shows that providing additional food resources does not necessarily improve food-management practices when the resources are poorly matched to household familiarity and practical capacity. Without targeted storage and preparation guidance, providing unfamiliar or excessive food may even generate additional waste [170].

The effectiveness of education and capacity-building depends largely on whether the promoted practices can be incorporated into everyday food routines. Sustained engagement, opportunities to practice new skills, and access to suitable planning, storage, and cooking tools influence whether knowledge is translated into repeated behavior [167,168]. Time pressure, convenience priorities, limited kitchen infrastructure, unfamiliar foods, and mismatches between educational advice and available resources may constrain implementation. Evidence regarding the long-term durability of these effects nevertheless remains limited [167,170].

Education and capacity-building should consequently be understood as one component of a broader food waste reduction strategy rather than as a stand-alone individual-level solution. Individual competence can be reinforced through accessible household tools, clearer date-label systems, suitable retail practices, and community or institutional programs adapted to consumers’ everyday circumstances. Businesses and public institutions can support this process by incorporating practical guidance into retail, foodservice, digital, and community settings and by ensuring that the surrounding food environment does not undermine the practices consumers are encouraged to adopt. Durable change is more likely when individual skills are supported by appropriate household resources, business practices, and institutional arrangements. Table 2 summarizes the five intervention categories and their key characteristics.

The intervention types discussed above address multiple barriers that consumers encounter when seeking to reduce food waste. Although these barriers are analytically distinct, they are closely interconnected in everyday food-related decision-making and include informational uncertainty, social expectations, decision environments, perceived trade-offs, and limitations in practical capacity. The effectiveness of these interventions depends on the behavioral mechanisms being targeted and on whether consumers encounter practical conditions that allow waste-reducing actions to be carried out. Table 3 synthesizes the mechanism-based linkages between the behavioral drivers identified in Section 3 and the intervention approaches reviewed in this section. These linkages are context-sensitive and should not be interpreted as fixed or one-to-one correspondences.

## 5. A Multi-Level Governance Perspective on Consumer Food Waste Reduction

**Figure 4:**
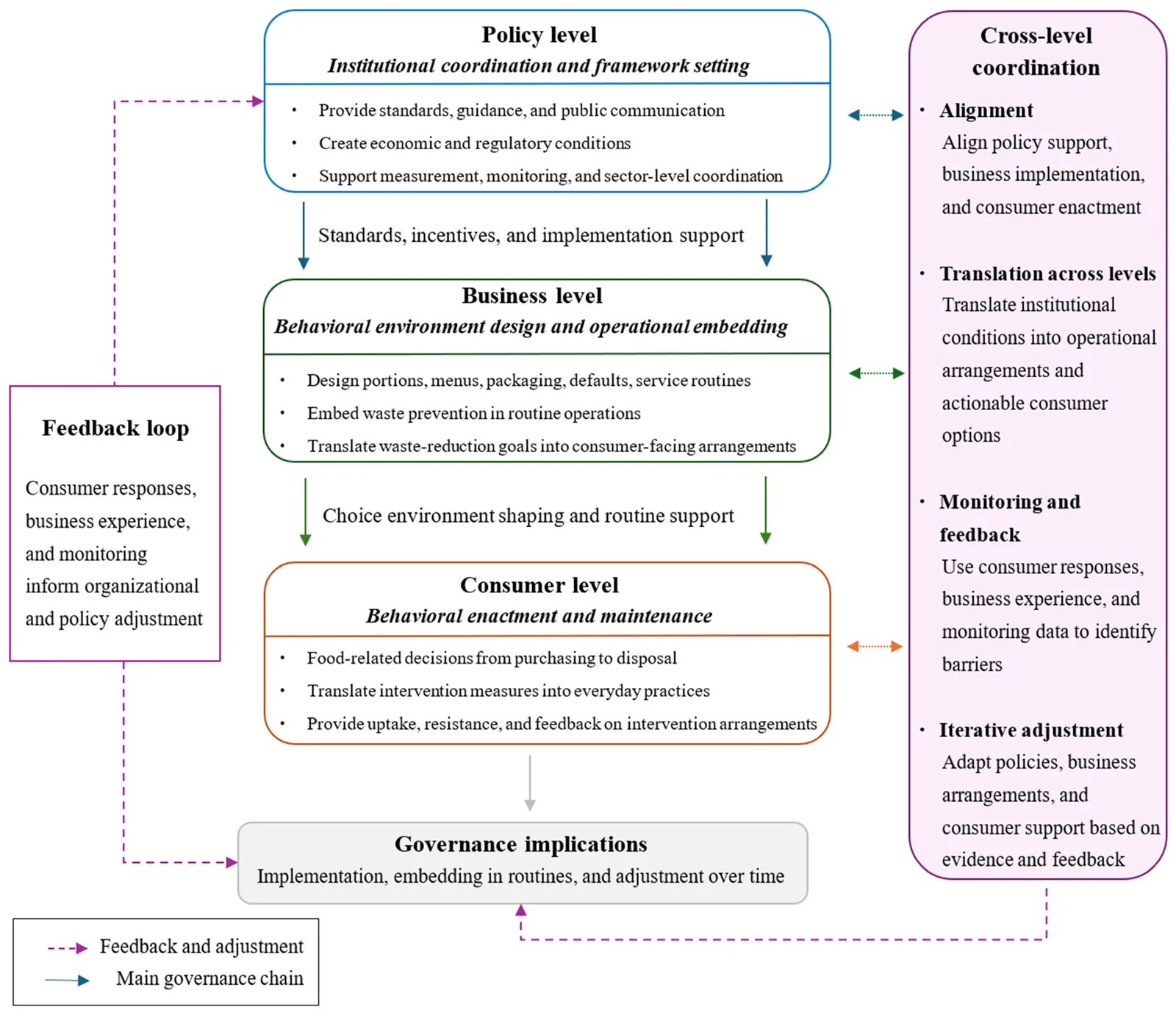
Multi-level governance framework for consumer food waste reduction.

The preceding sections have shown that consumer food waste arises from the interaction of cognitive biases, motivational drivers, and environmental and situational influences, and that behavioral interventions target different underlying mechanisms. However, whether these interventions can be effectively implemented and sustained over time depends not only on behavioral design but also on the governance conditions that support their adoption and integration into everyday practices. Reducing consumer food waste should therefore be understood as a multi-level governance issue rather than merely as a matter of individual behavior change. This review uses multi-level governance as an analytical perspective to examine how consumers, businesses, and policymakers perform distinct but interdependent functions in enabling and sustaining behavioral interventions. Consumers enact food-related practices through everyday routines, businesses embed waste-reduction goals in consumption environments and operational arrangements, and policymakers provide institutional, informational, and economic conditions that shape both consumer action and business implementation. The framework clarifies how interactions among these functions shape the conditions under which interventions can be implemented, embedded in routine practices, and adjusted over time. By moving beyond an exclusively consumer-centered explanation, this perspective helps identify how implementation responsibilities, enabling conditions, and coordination gaps are distributed across governance levels. Figure 4 summarizes these governance functions and their interrelationships.

### 5.1. Consumer Level

Consumers are the most immediate actors in food waste generation and prevention because decisions about purchasing, storage, portioning, consumption, leftover use, and disposal are ultimately enacted in everyday household and dining contexts. Their role is therefore to translate waste-reduction intentions and intervention measures into actual food-related practices. Consumers, however, should not be understood as fully autonomous decision-makers or as the sole source of responsibility for food waste [171]. As discussed above, their practices are shaped by cognitive biases, competing motivations, material conditions, social expectations, and the choice environments in which food-related decisions take place. The consumer level should therefore be understood primarily as the level at which waste-reduction practices are enacted and maintained, rather than as the sole locus of governance responsibility.

Consumer-facing interventions are more likely to be incorporated into everyday practices when they provide understandable, practical, and low-burden ways of acting. Timely prompts, practical guidance, supportive default options, or capacity-building measures may help consumers act on general intentions to reduce waste. The relevance of these measures depends not only on consumers’ willingness to change, but also on whether suitable alternatives are available at the points where purchasing, storage, ordering, consumption, and disposal decisions are made.

Consumers also engage in the governance process through their uptake of, resistance to, and feedback on waste-reduction arrangements. Their responses to portion options, packaging information, service routines, digital ordering interfaces, and policy measures can indicate whether an intervention is understandable, feasible, and acceptable in everyday contexts. Such feedback can help businesses and policymakers identify implementation barriers and adjust intervention strategies. Consumer action is therefore necessary but not sufficient for food waste reduction. Consumers ultimately carry out food-related practices, but the practical scope of their action is shaped by the information, options, infrastructures, and institutional conditions available to them.

### 5.2. Business Level

Within a multi-level governance framework, businesses and service providers occupy a distinctive position because they design many of the immediate settings in which food-related decisions are made. Retailers, restaurants, cafeterias, hospitality providers, and other foodservice actors influence consumer choices through portion sizes, menu structures, promotional practices, default options, service routines, food displays, and digital interfaces. Businesses can translate broad waste-reduction goals into concrete practices by incorporating food waste prevention into routine operations, such as menu planning, inventory management, portion design, serving procedures, staff training, and packaging design [172].

By modifying these operational and consumer-facing arrangements, businesses can reduce the behavioral burden placed on consumers. Flexible portion options, clearer ordering information, understandable date labels and storage instructions, and convenient leftover services can make lower-waste choices easier to identify and carry out. Such measures are particularly relevant when consumers support waste reduction in principle but face uncertainty, inconvenience, or a lack of feasible alternatives at the point of action.

However, business-level governance is also constrained by commercial considerations, such as revenue generation and customer experience [173,174]. Large portions, meal bundling, volume-based price promotions, extensive variety, and digital ordering promotions may increase sales or perceptions of value while also increasing the likelihood of over-ordering and surplus. Conversely, smaller portions or changes to promotional arrangements may be commercially sensitive when consumers interpret them as reducing value for money or service quality. Waste-reduction measures are therefore more likely to become embedded in routine business practice when they are compatible with operational processes, customer experience, internal performance systems, and relevant market and policy incentives.

Businesses should therefore be understood not merely as intermediaries between policy and consumers or as actors responsible for compliance, but as active designers and implementers of behavioral interventions. Their governance role lies in translating general waste-reduction objectives into concrete operational arrangements and consumer-facing choices. However, the sustained implementation of these measures depends on the alignment of internal management mechanisms, consumer responses, and the external institutional environment.

### 5.3. Policy Level

Governments and public institutions play an enabling and coordinating role in food waste governance, rather than directly determining the actions of consumers and businesses. Policymakers can influence the institutional, informational, and economic conditions under which food-related practices and business operations take place [175]. Policymakers can support food waste prevention by standardizing date-label terminology, food safety guidance, storage instructions, and anti-waste labels; clarifying liability rules and operational requirements for food donation; supporting waste-reduction education and capacity-building programs for consumers and businesses; requiring or encouraging businesses to measure, report, and disclose food waste; and applying economic and regulatory tools such as subsidies, rewards, charges, penalties, and compliance requirements.

These measures may influence consumer and business behavior through informational, incentive-based, or regulatory mechanisms. More importantly, they can clarify responsibilities, reduce uncertainty, align incentives, and establish more consistent expectations across consumers and businesses. Standardized information may make food labels easier for consumers to interpret and easier for businesses to communicate. Economic and regulatory instruments may alter the costs and benefits associated with waste prevention, while education and capacity-building programs can provide consumers and businesses with relevant knowledge and practical skills.

Policy intervention is particularly important where coordination problems cannot be resolved by individual actors. Consumers cannot standardize date labels or reshape market-wide information systems, and individual businesses may have limited incentives to adjust packaging, promotions, or portion design when such changes could affect sales or profitability. Policymakers can address these limitations by establishing common standards, providing sector-level guidance, supporting measurement and disclosure systems, and creating incentives for organizational implementation.

The existence of a policy measure does not, however, guarantee changes in everyday food-related practices. Standards, public communication, and economic instruments may have limited practical relevance when they are not translated into business routines and feasible consumer options. The policy level should therefore be understood as providing enabling conditions for waste-reduction practices, rather than as a substitute for organizational implementation or consumer action. Its effectiveness depends on how institutional arrangements are connected to business operations and everyday consumption practices.

### 5.4. Cross-Level Coordination

Within this framework, cross-level coordination refers to the alignment, translation, and adjustment of governance functions across the consumer, business, and policy levels [176,177]. Policy may reduce institutional uncertainty and provide shared standards, information, and incentives. Businesses may translate these conditions into operational routines, service arrangements, and consumer-facing choice environments. Consumers enact waste-related practices within these arrangements while accepting, resisting, adapting to, or providing feedback on their design. Coordination therefore involves more than the simultaneous participation of several actors; it concerns whether their respective functions reinforce one another during the implementation of behavioral interventions.

Date-label and storage-information interventions illustrate this relationship. Policy-level standardization may provide consistent terminology and safety guidance; businesses may communicate this information through packaging, retail displays, and consumer-facing prompts, and consumers must be able to understand and use this information in everyday food assessment and storage practices. Weakness at any point in this process may reduce the practical relevance of the intervention. Portion control and moderate ordering provide another example. Consumer willingness to avoid over-ordering may have limited practical effect when businesses do not offer flexible portion options or when promotional arrangements encourage excessive purchasing. Business-led changes may also be difficult to implement or maintain when they conflict with prevailing expectations regarding portion size, abundance, and value for money. Policy or sector-level guidance may help address these tensions by encouraging businesses to offer flexible portion options, improve menu and promotional practices, and test waste-reduction measures through pilot programs. Similarly, leftover takeaway and reuse depend on consumer acceptance, supportive service routines, available containers, and a social environment in which such practices are regarded as acceptable.

The importance of cross-level coordination becomes especially evident when governance functions are misaligned. Policy initiatives may remain symbolic when they are not translated into business routines and feasible consumer options. Business-led waste-reduction measures may be difficult to sustain when they conflict with revenue goals, service expectations, or internal performance priorities. Consumers may support waste reduction but remain unable to change or sustain their practices when they lack accessible portion options, takeaway support, suitable storage conditions, or other practical alternatives.

Cross-level coordination should therefore be understood as a dynamic process of adjustment rather than a linear process of top-down governance. Policy frameworks shape the conditions for business implementation, businesses structure consumers’ immediate decision environments, and consumer responses provide information that may support further organizational and institutional adjustment. Monitoring data, consumer experience, and market responses can help identify where intervention arrangements are unclear, impractical, or inconsistent with established routines. From this perspective, the framework suggests that cross-level coordination shapes the conditions under which behavioral interventions may be implemented, embedded in routine practices, and adjusted over time.

## 6. Discussion

Drawing together the preceding analysis, this discussion considers the broader implications of the review for understanding and addressing consumer food waste. It first examines how behavioral drivers, intervention strategies, and governance conditions are connected, then considers the implications of this relationship for intervention selection and implementation. The discussion further identifies the main temporal, contextual, and structural boundaries of the available evidence before outlining the limitations of the review and priorities for future research.

### 6.1. Linking Behavioral Drivers, Intervention Design, and Governance

The main theoretical implication of this review lies in linking behavioral drivers, intervention strategies, and governance conditions within a shared analytical framework. The behavioral drivers reviewed in this article indicate that consumer food waste emerges from recurring food-related decisions shaped by the interaction of cognitive biases, motivational drivers, and environmental and situational influences. These decisions may involve uncertainty, competing priorities, practical constraints, and immediate consumption cues. Similar waste outcomes may therefore emerge through different behavioral pathways, indicating that food waste cannot be adequately explained by insufficient awareness or individual responsibility alone.

This linkage also clarifies the role of behavioral interventions. Information, social norms, choice architecture and environmental design, incentives, and education or capacity-building address different but interconnected barriers by improving the usability of information, reshaping social expectations and immediate choice environments, altering perceived trade-offs, and strengthening practical capacity. Their relationship with behavioral drivers is not one-to-one. A single intervention may address several mechanisms, while the same barrier may require different responses depending on the decision context. The intervention typology therefore identifies the principal behavioral logic through which change may be encouraged, rather than assigning each behavioral driver to a fixed intervention tool.

Behavioral fit alone, however, does not ensure that an intervention can be translated into practice. Implementation also depends on whether consumers have feasible opportunities to act, businesses can incorporate waste-reduction measures into operational and consumer-facing arrangements, and policy conditions provide appropriate institutional, informational, and economic support. The multi-level governance perspective adds this implementation dimension by locating relevant responsibilities and constraints across consumer, business, and policy levels. Taken together, behavioral drivers explain why food waste occurs, intervention strategies indicate how change may be encouraged, and governance conditions shape whether these measures can be implemented and embedded in actual consumption practices.

### 6.2. Implications for Governance Practice

Building on this integrated understanding, this review offers several implications for the governance practice of consumer food waste reduction, particularly for how behavioral interventions are selected, combined, and implemented. The first practical implication is that intervention selection should begin with a diagnosis of the waste-generating situation rather than with a preferred intervention tool. Different waste-generating situations, such as date-label confusion, over-acquisition, reluctance to use leftovers, or poor household food management, may require different forms of support that involve different behavioral barriers and should not automatically be addressed with the same intervention tool. The choice of intervention should therefore be informed by the targeted behavior, the mechanisms contributing to it, and the setting in which the relevant decision occurs.

Second, interventions may be combined when several barriers operate together, but adding more components does not necessarily produce stronger effects. Information, social norm messaging, choice architecture, incentives, and education or capacity-building measures address different aspects of food-related behavior. In some cases, a targeted informational prompt may be sufficient to reduce uncertainty at the point of decision. In others, information may need to be supported by feasible portion options, convenient leftover management, practical skills, or socially acceptable service routines. The crucial question is not whether interventions are used alone or in combination, but whether the selected measures address the main barriers that prevent waste-reducing action in that context.

Third, interventions should be implemented at meaningful decision points. Information needs to be understandable, timely, and linked to an actionable option; social norm messages need to refer to credible reference groups and clearly specified behaviors, while avoiding the unintended normalization of wasteful practices; choice architecture needs to fit service routines and consumer expectations rather than creating excessive inconvenience or dissatisfaction; incentives need to avoid excessive burdens, perceptions of coercion, or unintended responses; and education and capacity-building measures need to provide skills that can be incorporated into consumers’ everyday routines. These conditions suggest that governance practice should pay attention not only to how an intervention is designed, but also to whether consumers and organizations have realistic opportunities to implement and sustain the intended action.

### 6.3. Challenges and Boundary Conditions of Behavioral Interventions

The effects of behavioral interventions depend not only on their design, but also on the temporal, contextual, and structural conditions under which they are implemented. Clarifying these boundary conditions is important for assessing how intervention findings should be interpreted and applied.

The duration of the intervention effect is an important boundary condition. Many interventions can influence immediate decisions, but their effects may weaken after the intervention is removed or consumers return to established routines [164,178]. Continued behavior change may be more likely when waste-reducing practices can be incorporated into repeated food-related routines, although evidence on sustained reductions in actual food waste remains limited.

Contextual variation also limits the transferability of interventions. Food waste does not occur in a uniform environment, and findings from one setting cannot be assumed to apply directly to another [22]. Household routines differ from foodservice practices, online ordering differs from in-store purchasing, and group dining differs from individual consumption. Cultural expectations regarding hospitality, abundance, thrift, and leftover use may also affect how consumers interpret waste-reduction messages or service arrangements. External validity therefore remains an important challenge: an intervention that appears effective in one setting may produce weaker, different, or unintended effects when transferred to another.

A further boundary condition relates to the allocation of responsibility. Behavioral interventions may place excessive responsibility on consumers when their scope is narrowly focused on individual decision-making, while organizational and structural conditions that shape food-related practices receive less attention. Retail promotions, portion sizes, digital ordering interfaces, storage infrastructure, and service processes may influence waste before consumers explicitly decide whether to discard food. The effectiveness of consumer-facing interventions therefore depends partly on whether they are supported by changes in the environments in which food-related decisions take place.

### 6.4. Limitations and Future Research Directions

The existing literature has several limitations that influence the current understanding of consumer food waste and behavioral interventions. Evidence remains uneven across behavioral mechanisms, intervention types, consumption settings, and food categories. Many studies focus on specific interventions or isolated behavioral factors, while less is known about how multiple behavioral mechanisms interact with situational conditions and food-specific characteristics in everyday food practices. Research has also concentrated largely on household and conventional foodservice settings, with comparatively less attention to retail environments, online food delivery platforms, culturally diverse consumption contexts, and differences among food categories. In addition, much of the intervention literature focuses on short-term behavioral responses, while the organizational processes and implementation conditions that influence whether interventions become part of routine practice remain less developed.

This review also has some limitations. First, the structured literature search relied solely on the Web of Science Core Collection, without simultaneously searching other databases such as Scopus and Google Scholar. Since different literature sources vary in terms of journal coverage, disciplinary focus, and geographic scope, relying on a single database may result in certain coverage biases, leading to the failure to identify or include some relevant studies, which in turn affects the comprehensiveness and representativeness of this review. Although this review supplemented the database search with forward and backward citation tracking, this approach still cannot completely eliminate potential omissions resulting from reliance on a single data source. Second, the review limited the search scope to English-language, peer-reviewed journal articles published between January 2010 and January 2026; therefore, it may have omitted earlier foundational research in this field, literature published in other languages, and relevant gray literature. Third, the search strategy was based on predefined search terms; thus, relevant studies using different concepts or technical terminology may not have been fully identified. Fourth, as a narrative review, the classification of the literature inevitably involved a degree of subjective judgment. Many studies addressed multiple behavioral drivers or intervention types, and assigning them to a principal analytical category was not always straightforward. In addition, the methodological quality of the included studies was not assessed using a formal appraisal tool, which limits the extent to which the methodological robustness of the evidence can be systematically compared across studies. Differences in research design, intervention duration, consumption setting, measurement method, and outcome definition further limit direct comparison across studies and make a definitive ranking of intervention effectiveness difficult.

Finally, the reviewed evidence also provides limited direct comparison between interventions operating primarily at a single governance level and those supported across consumer, business, and policy levels. The proposed contribution of cross-level coordination to the implementation, embedding, and adjustment of behavioral interventions should therefore be treated as an analytical proposition requiring further empirical examination.

Building on these limitations and the broader gaps in the existing evidence base, future research could advance the field in several directions. First, longitudinal studies, repeated field interventions, and follow-up measurements are needed to examine whether immediate behavioral responses develop into sustained changes in actual food waste. Research should also pay greater attention to how interventions are incorporated into household routines, business operations, service procedures, and digital systems after their initial introduction.

Second, food waste measurement should increasingly combine self-reported information with methods closer to observed behavior, including food waste diaries, waste audits, direct weighing, plate-waste observation, purchase and disposal records, digital tracking, and smart-bin data. More consistent reporting of food categories, participant characteristics, intervention duration, consumption setting, and outcome definitions would improve comparability and help distinguish changes in awareness or intermediate behavior from reductions in actual discarded food.

Third, more mechanism-sensitive and context-sensitive research is also needed. Future studies should examine not only whether an intervention produces an effect, but also which behavioral mechanisms it influences, for whom, and under what conditions. More attention should be given to online food delivery and other platform-mediated environments, where interface design, default options, recommendations, and ordering thresholds may generate waste pathways that differ from those found in households or conventional foodservice settings. Cultural expectations concerning hospitality, abundance, thrift, freshness, value for money, and leftover use should also be examined alongside food-specific characteristics such as perishability, safety risk, storage requirements, and symbolic value.

Finally, research should examine the organizational and governance conditions surrounding intervention implementation. Studies could investigate how retailers, foodservice, hospitality, and digital platforms translate waste-reduction goals into operational routines and consumer-facing choice environments. Comparative case studies, field experiments, and mixed-methods research could further assess how consumer uptake, business implementation, and policy support interact. Direct comparison of interventions operating at different governance levels would help clarify whether, how, and under what conditions cross-level coordination influences implementation continuity and actual food waste outcomes.

## 7. Conclusions

This review has examined how consumer food waste is shaped by behavioral drivers, intervention strategies, and governance conditions. It shows that consumer food waste cannot be attributed solely to insufficient awareness or individual behavior. Rather, it emerges from the interaction of cognitive biases, motivational drivers, environmental and situational influences, and the consumption environments in which everyday food-related practices take place. Behavioral interventions can help reduce food waste when they are designed to address relevant barriers and embedded at meaningful decision points, but their effectiveness varies according to context and the extent to which waste-reducing practices can be maintained over time. The multi-level governance perspective developed in this review further highlights how consumer action, business-designed choice environments, and policy-level support jointly shape the conditions under which interventions are implemented and embedded in practice. By linking behavioral drivers, intervention strategies, and governance conditions, this review provides an integrative perspective for understanding the conditions under which consumer food waste reduction may move beyond isolated interventions toward more coordinated and adaptive governance practices.

## Figures and Tables

**Table 1:** Behavioral drivers of consumer food waste.

Main Category	Specific Driver	Behavioral Mechanisms Suggested by the Literature	Typical Waste Pathway	Potential Intervention Directions	References
Cognitive biases	Underestimation of waste and its consequences	Consumers perceive their waste as less frequent, extensive, or consequential than it actually is	Weak urgency and limited engagement in waste-prevention practices	Make personal food waste and its consequences more visible and provide actionable guidance	[40,41,43,44,45,46,50]
	Date-label misunderstanding	Consumers may treat quality-related dates as fixed safety deadlines and adopt precautionary discard decisions under uncertainty	Premature disposal of edible food	Standardized terminology, clear safety–quality distinctions, and food-specific sensory and storage guidance	[37,52,55,56,57,58,60,61,62,63,64]
	Overconfidence and optimism bias	Consumers overestimate their future consumption, food-related knowledge, and food-assessment ability	Over-purchasing, reduced engagement with corrective information, and premature disposal	Calibration feedback, planning support, reminders	[33,66,67,68,69]
	Biased food management practices	Small errors accumulate across planning, purchasing, storage, cooking, and reuse	Over-purchasing, forgotten or spoiled food, over-preparation, and discarded leftovers	Inventory tools, storage guidance, meal planning, cooking and reuse skills	[36,71,72,73,74,75,76,80,81,82]
Motivational drivers	Convenience orientation	Consumers minimize time, physical effort, and mental effort	Weak planning, over-ordering, limited leftover reuse	Make prevention convenient; flexible portions and bundles	[88,92,93,94,95,96]
	Hedonic motivation	Consumers prioritize freshness, variety, sensory appeal, and immediate enjoyment	Over-selection and rejection of less appealing leftovers	Align waste reduction with enjoyment, quality, and pleasurable eating experiences	[89,100,101]
	Social image motivation	Food is used to display generosity, hospitality, identity, or social status	Over-ordering and reluctance to pack leftovers	Image-based framing and supportive social norms	[102,103,104,105,106]
Environmental and situational influences	Physical and decision environment cues	Plate size, service format, menu design, and promotions shape quantity judgments	Excessive serving, purchasing, or ordering	Choice architecture, portion and menu design, service and promotion redesign	[110,111,112,113,115,116,117,118]
	Domestic storage infrastructure	Visibility, organization, temperature, packaging, and storage space affect monitoring and preservation	Forgotten or overstocked food, delayed use, and spoilage	Storage organization, packaging and preservation support	[78,119,120,121,122,123,124]
	Sociocultural factors	Norms of abundance, care, hospitality, and role expectations shape appropriate provision and leftover handling	Overprovisioning, socially normalized surplus, and reluctance to take leftovers away	Reframe moderate provision and leftover reuse as responsible care	[109,125,127,128,129,130]

**Table 2:** Types of behavioral interventions for reducing consumer food waste.

Intervention Category	Core Intervention Logic	Illustrative Intervention Configurations	Key Conditions and Limitations	Governance Relevance	References
Information-based interventions	Reduce uncertainty and improve information usability at relevant decision points	Date-label clarification combined with safety–quality distinctions and sensory-assessment guidance; portion references and actionable prompts embedded in menus, tables, buffet displays, packaging, or storage locations	Timely, concrete, understandable, and actionable information; sufficient confidence in interpretation; visibility alone may be insufficient; limited evidence of effects after prompts are removed	Policy standards for labeling and consumer information; business embedding in menus, packaging, retail displays, digital interfaces, and storage environments	[13,14,15,131,132,133,134,135,136,137,138]
Social norm interventions	Reshape perceptions of what relevant others commonly do and which behaviors are socially approved	Descriptive or injunctive norm messages linked to moderate ordering or leftover takeaway; normative prompts combined with portion signage, measuring tools, or practical household guidance	Credible reference groups; behavior-specific and culturally appropriate framing; avoidance of messages that normalize waste; norm effects may be difficult to separate from accompanying prompts or tools	Restaurants, retailers, institutions, digital platforms, and public communication reinforcing the social acceptability of waste-reducing practices	[140,141,142,143,144,145,146,147,148]
Choice architecture and environmental design interventions	Restructure immediate decision environments so that lower-waste choices become easier or more salient	Adjustable portion options; smaller tableware and menu-based portion design; self-service and tray arrangements; visible and convenient leftover takeaway provision	Fit with food characteristics, cultural context, service routines, and consumer expectations; preservation of autonomy and satisfaction; takeaway uptake does not necessarily demonstrate actual waste reduction	Restaurants, foodservice providers, retailers, schools, and other institutions play a central role in designing the immediate food-choice environment	[112,118,152,153,154,155,156]
Incentive-based interventions	Alter consumers’ perceived costs and benefits associated with food-related decisions	Unit-based or pay-by-weight charging combined with flexible quantity choice; side-dish pricing, discounts, rewards, or penalties accompanied by feasible lower-waste alternatives	Clear connection between incentives and target behavior; sufficient consumer control and feasible alternatives; monitoring for overconsumption, counterproductive responses, and customer-retention risks	Government and municipal economic signals translated by businesses into workable pricing, ordering, portion, and reward arrangements	[115,158,159,160,161,162,163,164]
Education and capacity- building interventions	Strengthen consumers’ practical knowledge, skills, confidence, and routines for everyday food management	Tailored action knowledge combined with goal setting or commitment; meal-planning, shopping, storage, freezing, portioning, and leftover-use skills supported by household tools and reminders	Relevance to household circumstances, time, food familiarity, and available infrastructure; opportunities to practice skills; improved competence may not produce measurable waste reduction; limited long-term evidence	Public institutions, communities, and businesses providing practical resources and supportive environments; consumers integrating skills into everyday routines	[47,165,166,167,168,170]

**Table 3:** Mechanism-based linkages between behavioral drivers and intervention strategies.

Behavioral Driver	Core Mechanism or Barrier	Relevant Intervention Approaches	Mechanism-Based Linkage
Underestimation of waste and its consequences	Consumers underestimate the magnitude and consequences of their own food waste	Information-based interventions; education and capacity-building	Greater visibility corrects underestimation, while practical guidance converts awareness into routine action
Date-label misunderstanding	Quality-related labels are treated as safety deadlines under uncertainty and risk aversion	Information-based interventions; education and capacity-building	Label clarification reduces uncertainty, while sensory-assessment skills support more confident edibility judgments
Overconfidence and optimism bias	Consumers overestimate future consumption, planning reliability, knowledge, and food-assessment ability	Information interventions; education and capacity-building	Calibration feedback exposes forecasting errors, while capability-building strengthens the planning and management skills needed to act on more realistic estimates
Biased food-management practices	Small errors accumulate across planning, purchasing, storage, preparation, and leftover reuse	Education and capacity-building; information-based interventions	Practical skills address recurring management errors, while timely prompts reinforce practices at decision points
Convenience orientation	Consumers seek to minimize time, physical effort, and cognitive effort	Choice architecture and environmental design; information-based interventions; education and capacity-building	Redesigning friction makes lower-waste choices easier and excessive selection less convenient; information and skills remain useful only when easy to apply
Hedonic motivation	Preferences for freshness, variety, sensory appeal, and immediate enjoyment may encourage over-selection or reduce leftover desirability	Choice architecture and environmental design; incentive-based interventions in selected contexts	Experience-compatible design preserves satisfaction, while incentives may offset lower perceived appeal in selected contexts
Social image motivation	Generosity, hospitality, identity, and face concerns may encourage over-ordering and discourage takeaway	Social norm interventions; information-based interventions; choice architecture and environmental design	Norms make moderate ordering and takeaway socially acceptable, while prompts and convenient services enable action
Physical and decision environment cues	Portions, menus, promotions, defaults, tableware, and service formats shape quantity judgments and selection	Choice architecture and environmental design; information-based interventions; incentive-based interventions	Environmental redesign acts before surplus occurs, while information and economic cues improve quantity decisions
Domestic storage infrastructure	Visibility, organization, packaging, temperature, and storage space affect monitoring and preservation	Information-based interventions; education and capacity-building	Prompts and skills improve existing storage practices but cannot resolve physical infrastructure constraints
Sociocultural factors	Norms of abundance, care, hospitality, thrift, and role responsibility shape appropriate food provision	Social norm interventions; information-based interventions; choice architecture and environmental design	Norms reframe moderate provision and reuse, while feasible portion and takeaway options support enactment

## Data Availability

No new data were created or analyzed in this study. Data sharing is not applicable to this article.
